# Extramedullary myeloma in an HIV-seropositive subject. Literature review and report of an unusual case

**DOI:** 10.1186/1746-160X-5-4

**Published:** 2009-01-20

**Authors:** Liviu Feller, Jason White, Neil H Wood, Michael Bouckaert, Johan Lemmer, Erich J Raubenheimer

**Affiliations:** 1 Department of Periodontology and Oral Medicine, School of Dentistry, University of Limpopo, Pretoria, South Africa; 2 Department of Maxillofacial and Oral Surgery, School of Dentistry, University of Limpopo, Pretoria, South Africa; 3 Department of Oral Pathology, School of Dentistry, University of Limpopo, Pretoria, South Africa

## Abstract

Myeloma is characterized by monoclonal bone marrow plasmacytosis, the presence of M-protein in serum and/or in urine and osteolytic bone lesions. HIV-seropositive subjects with myeloma are younger at the time of diagnosis of the tumour and usually the myeloma has a more aggressive clinical course than it does in HIV-seronegative subjects.

A case of an HIV-seropositive woman in whom myeloma was diagnosed following progressive swelling of the face, is reported. In addition to bone marrow plasmacytosis and the presence of M-protein in the serum, the patient had an extramedullary lesion affecting the oral cavity, maxilla, parotid gland and paranasal sinuses, and extending intracranially and intraorbitally.

## Background

Myeloma is an incurable haematological malignancy, the characteristic cell type of which is terminally differentiated B-lymphocytes. The affected cells accumulate in the bone marrow, and myeloma accounts for about 10% of all haematological cancers. Myeloma affects both the immune and skeletal systems and the tumour cells have cytogenetic alterations in the variable regions of immunoglobulin (Ig) heavy and light chain genes. These cytogenic abnormalities may mediate the uncontrolled proliferation, prevent the differentiation, and contribute to the prolonged survival of myeloma cells [1,2].

Only 5% of subjects with myeloma go into remission after treatment and the median survival time is about 3 years. The incidence of myeloma increases with age and the median age at diagnosis is 68. Males are affected more frequently than females and black persons are affected twice as frequently as whites. In the late stages of myeloma disease, increasing numbers of plasma cells may be detected in the circulating blood and skeletal extramedullary myeloma tumours may develop with increasing frequency [1-4].

The uncontrolled proliferation of myeloma cells is accompanied by an increase in their production of monoclonal Ig proteins (M-protein). The presence of M-protein in serum or urine can be detected by electrophoresis, and immunoelectrophoresis or immunofixation is used to identify the specific heavy (M, G, A, D, E) and light (κ or λ) Ig chain class [3].

Some or all of the following criteria would constitute evidence for the diagnosis of myeloma: evidence of M-protein in the serum or urine (usually ≥ 30 g/L); at least 10% plasma cells on a myelogram; demonstration of monoclonal plasma cells on bone marrow biopsy; and end-organ damage that may be hypercalcaemia, renal insufficiency, anemia, osteolytic bone lesions or extramedullary dissemination of myeloma tumour cells [3,5].

Over 90% of subjects with myeloma have M-protein in the serum or in the urine at the time of diagnosis, about 60% of them ≥ 30 g/L. Monoclonal plasma cells usually account for ≥ 10% of all bone marrow nucleated cells, but may range from ≤ 5% to almost 100% [5,6]. At the time of diagnosis, subjects with myeloma may present with hypercalcaemia (15–20% of subjects); with renal insufficiency measured as serum creatin > 173 mmol/l (about 20%); normocytic normochromic anemia (about 60%); and bone lesions or pathological fractures of bone (about 80%) [5]. The occurrence of extramedullary dissemination of myeloma tumour cells at the time of diagnosis is uncommon.

Solitary plasmacytoma (SP) is a localized variant of myeloma presenting either as solitary bone plasmacytoma (SBP), or as extraskeletal soft tissues when it is termed extramedullary plasmacytoma (EMP) [7,8].

SP is less common than myeloma and affects younger subjects who have a median survival of 10 years or more [2]. The diagnosis of SP is based on histological demonstration of monoclonal proliferation of plasma cells without evidence of end-organ damage. Generally subjects with SP do not have M-protein in the serum or in the urine, and do not have monoclonal plasmacytosis of bone marrow [9].

SBP is more common in males than in females, most commonly affects the axial skeleton, and the onset is about 10 years earlier than myeloma. In SBP there is sometimes evidence of M-protein < 20 g/L in the serum and/or in the urine, and monoclonal plasmacytosis of the bone marrow of < 5% [9-11]. About 50% of subjects with SBP will develop overt myeloma some 2–3 years after treatment of their SBP [5,12,13].

In contrast to SBP, EMP most frequently involves the submucosal lymphoid tissue of the paranasal sinuses, nasopharynx or the tonsils; [3,11,14] EMP is less common than SBP and occurs in slightly older subjects [13]. About 15% of subjects with EMP progress to myeloma following treatment, but the rest are cured [12]. The prognosis of EMP is therefore substantially different to that of SBP and of myeloma, suggesting some difference in the pathogenic mechanisms of the 3 diseases [8].

Myeloma needs to be differentiated from other monoclonal gammopathies including heavy chain disease, monoclonal gammopathy of undetermined significance, Waldenström macroglobulinemia, SP, plasma cell leukemia and plasmablastic lymphoma [3].

## Case presentation

**Figure 1:**
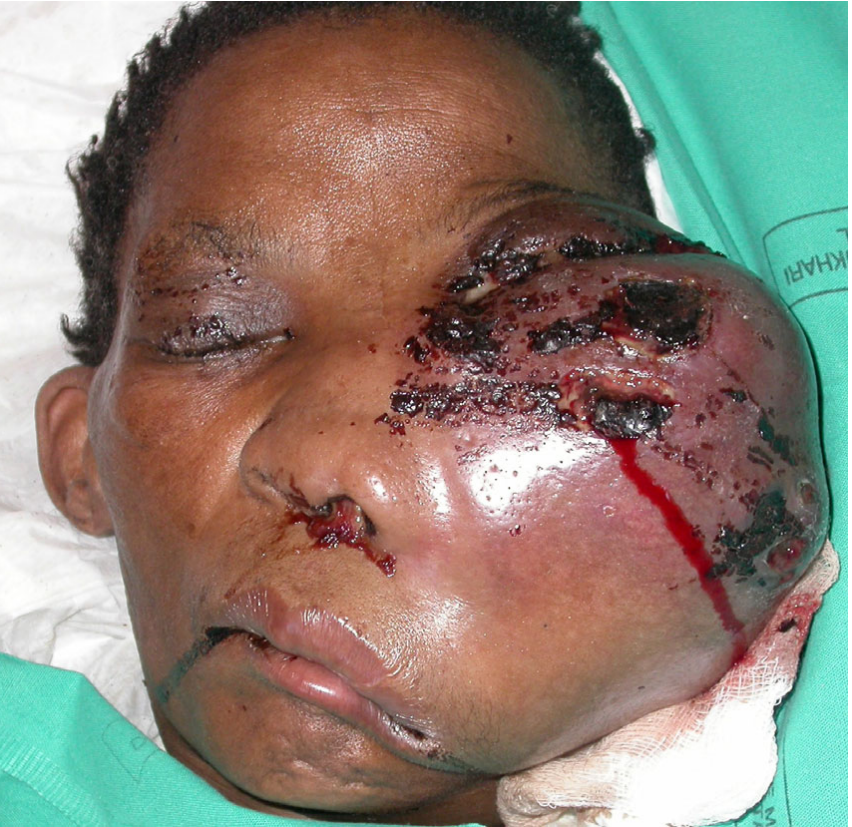
**Extramedullary myeloma of the head and face. Note the marked distortion of the nose, lips and left eye. The tumour affects the left maxillary and zygomatic area. There was a midline shift of the nose and the chin**.

**Figure 2:**
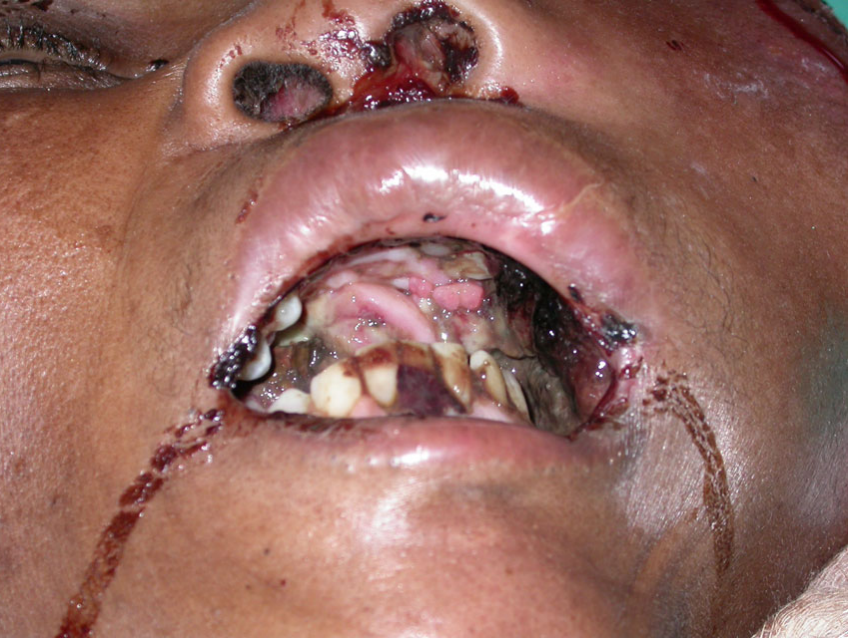
**Note the exophytic, irregular lesions protruding from the palate and from the left nostril**.

A 48 year-old black female presented at the Medunsa Oral Health Center with a large swelling of the left side of her face, and proptosis of her left eye (figure 1). The facial mass was firm and immobile. The nasolabial furrow was obliterated by the swelling, the nose was displaced to the right and the tumour was fungating from the left nostril. Intraorally, there was a large soft tissue mass growing from the maxilla, and extending from the left side across the midline. A large portion of this mass was necrotic (figure 2). The patient stated that the facial swelling had rapidly enlarged over the previous month, and that she had recently lost the sight of her left eye. Because of language and cultural difficulties in communication, we could not determine why she had not received medical attention before our consultation.

**Figure 3:**
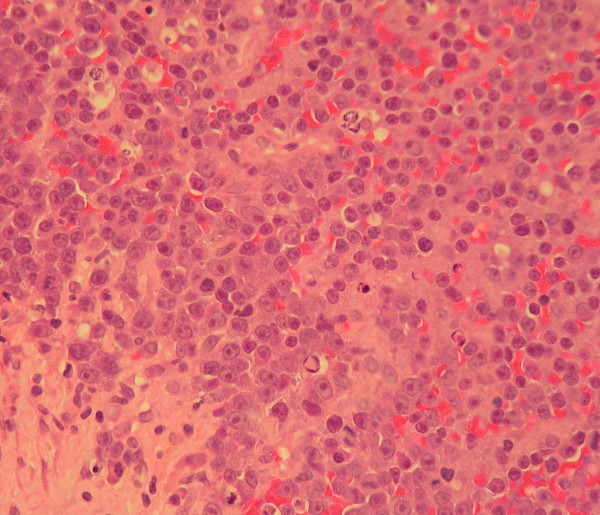
**High power photomicrograph of the myelomatous infiltrate. Several plasmablasts with prominent nucleoli are present (H&E stain, ×400)**.

Microscopic examination of a biopsy specimen from the intraoral mass showed a neoplastic plasma cell tumour. The densely packed tumour cells were mainly plasmablasts with prominent nucleoli (figure 3). Aspiration of bone marrow from the femur demonstrated plasmacytosis (9% of the myelogram) without an increased number of blasts. The plasma cells were preponderantly mature with occasional binucleated forms. A trephine biopsy of bone marrow was not done, so the clonality of the plasma cell population could not be determined. Erythropoiesis was reduced.

Serum protein electrophoresis and immunofixation showed IgG kappa monoclonal protein. Both serum IgG (35.89 g/L, reference range 7.00 – 16.00) and kappa light chain (8.4 gr/L, reference range 0.6 – 1.3) were elevated. The patient was HIV-seropositive with a CD4+ T cell count of 41 × 10^-6^/L, and the percentage of CD4 lymphocytes was 9.06. There was normocytic normochromic anemia, lymphopenia, and a high platelet count. Serum calcium and creatin levels were normal. A skeletal survey excluding the head showed no abnormalities. Ophtalmological examination confirmed blindness of the patient's left eye.

**Figure 4:**
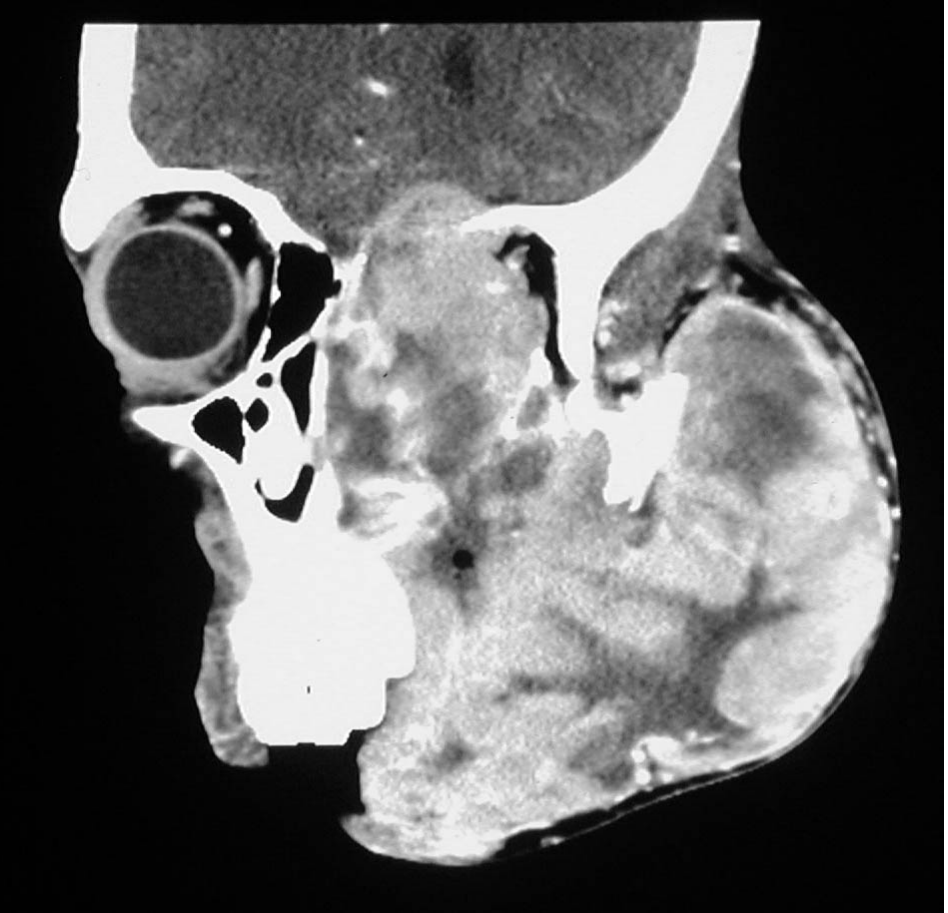
**Sagittal CT cut showing the extensive destruction of the left maxilla, and the left paranasal tissues. The left orbit is filled with tumorous tissue**.

**Figure 5:**
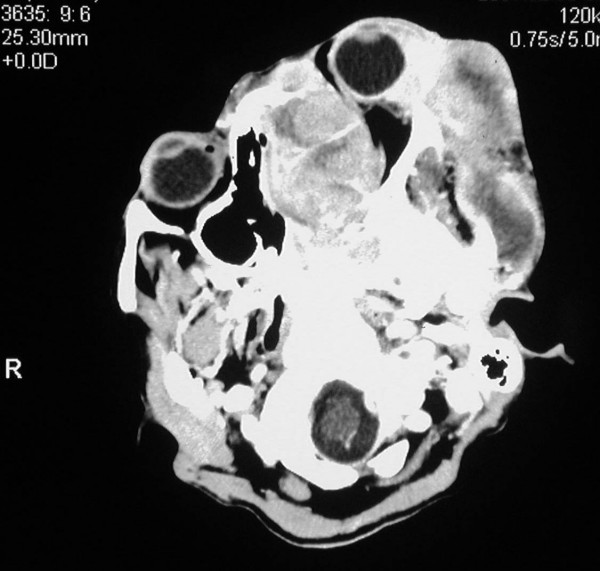
**Axial CT cut demonstrating invasion of the tumour into the left orbit, causing proptosis; and the extension of the tumour to the left ethmoidal sinus and the temporal and infra-temporal fossae**.

**Figure 6:**
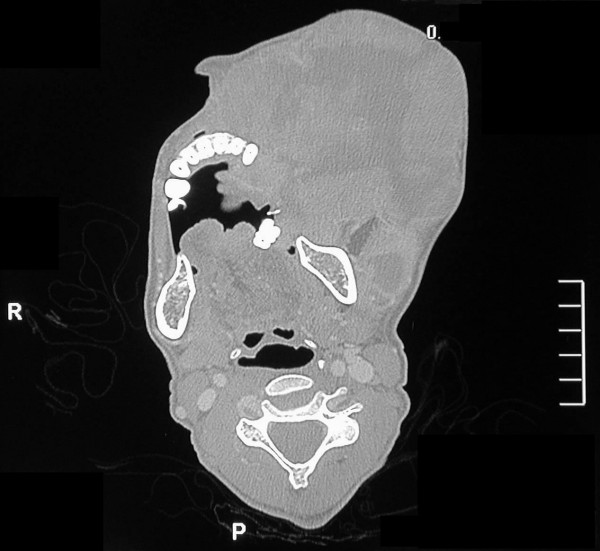
**Axial CT cut showing the invasion of the floor of the mouth by the tumour**.

**Figure 7:**
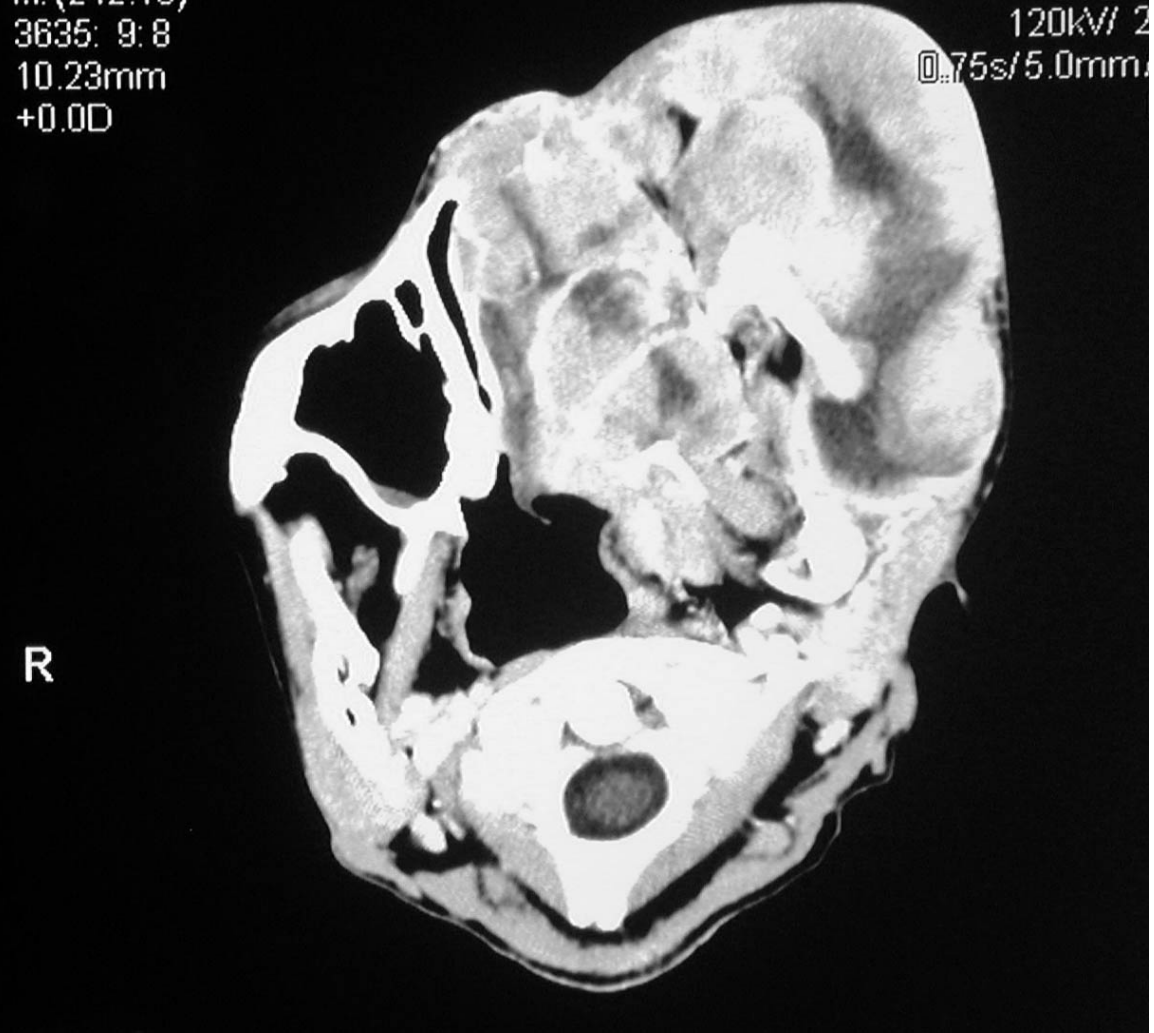
**Axial CT cut showing the extent of the heterogeneous tumour**.

Computed tomography revealed a large soft tissue mass measuring 12 cm × 12 cm that had caused destruction of the left maxillary ethmoid sinus, the sphenoid sinuses and the left nasal cavity, and extended intra-cranially into the anterior cranial fossa (figure 4) The intra-orbital tumour mass caused severe proptosis of the left eye (figure 5). The mass involved the left nasopharynx, as well as the parotid and masseteric spaces and the buccal tissues on the left side. The left submandibular space and the floor of the mouth were also affected by the tumour (figure 6). The mandible appeared normal but had been displaced, without evidence of osteolytic or sclerotic lesions. The tumour mass appeared heterogeneous with hypodense areas of necrosis (figure 7). Bilateral enlarged cervical lymphnodes were evident at various levels of the radiographic cuts.

A diagnosis of myeloma was made and the patient was referred to the regional hospital where she died 5 days later from respiratory complications.

## Discussion

The common denominator to myeloma and SP is the uncontrolled proliferation of myeloma cells. While myeloma is characterized by monoclonal plasmacytosis of the bone marrow, with or without bone destruction, and by the presence of M-protein in urine or in serum, in SP there is no evidence of significant bone marrow plasmacytosis and less than 30% of subjects with SP have M-protein, and when it is present it is low compared to the level in myeloma [3,7,8].

Our patient met the criteria for a definite diagnosis of myeloma: her bone marrow plasma cell count was increased (9% of the myelogram) which approaches the lower limits for myeloma (10%); the serum protein quantification and immunofixation showed an increased level of IgG kappa monoclonal protein (M-protein); the presence of an extramedullary myeloma tumour; and she had a normocytic normochromic anemia associated with decrease in bone marrow erythropoiesis.

Since the bone marrow plasma cells were preponderantly mature forms, and since their clonal nature had not been established, it is possible that the bone marrow plasmacytosis was reactive to HIV. Even if the bone marrow plasmacytosis was not associated with the myeloma disease, the criteria listed in the previous paragraph are sufficient to establish a diagnosis of myeloma.

Extramedullary dissemination of myeloma usually occurs several years after the initial diagnosis of myeloma, but sometimes the extramedullary myeloma can be present at the time as the diagnosis of myeloma [7]. In the present case it could not be determined whether the extramedullary mass developed concurrently with the myeloma, subsequently to the primary myeloma or whether this was a primary extramedullary plasmacytoma that then progressed to a frank myeloma.

EMP in subjects with myeloma is not a rare finding [15]. However, EMP that occurs concurrently with myeloma should be regarded as extramedullary myeloma (EMM), and EMP and EMM are two distinct entities with different prognoses.

Subjects with EMP do not usually have M-protein in serum and/or in urine, but when present it is only in low levels; nor do they have bone marrow plasmacytosis and their skeletal survey is normal. Less than 30% of subjects with EMP progress to myeloma and 70% of these subjects survive 10 years [9]. In contrast, EMM is a dissemination of myeloma cells and should be regarded as end-organ damage. Subjects with EMM manifest the laboratory characteristics of myeloma, therefore the prognosis of subjects with EMM is worse than the prognosis of subjects with EMP, and the management of the two entities differs.

Although, there is a theoretical possibility of concurrence of EMP with myeloma, this is of academic interest only because the much more serious myeloma demands priority.

Early stages of B cell maturation occur in the bone marrow and are regulated by signals from bone marrow stromal cells. In the bone marrow, the B cell differentiates up to the expression of cell surface Ig (s-Ig) receptors. At this point the B cells exit the bone marrow into the peripheral circulation and migrate to secondary lymphoid organs, including lymph nodes, spleen and Peyer's patches of the gut. In the germinal centers of the peripheral lymph tissue, further differentiation of B cells is mediated by antigen-specific interaction with B cell s-Ig that leads to Ig gene rearrangement and a switch from the expression of IgM to the expression of IgG or IgA [16]. These activated B cells (plasmablasts) exit into the bone marrow, stop proliferating and differentiate into Ig-secreting plasma cells. The homing of plasma cells into the bone marrow is mediated by adhesion molecules and interleukins mainly IL-6 [1].

The monoclonal precursors of myeloma cells in the bone marrow originate in the lymph nodes. The mechanisms that enable these precursor cells to selectively lodge in the bone marrow where the particular microenvironment is conducive to their differentiation, proliferation and survival are not well understood. However, it is probable that the bone marrow microenvironment provides the specific chemotactic signals, and the monoclonal myeloma precursor cells express the necessary cell surface receptors for the bone marrow lodgement. There is adhesion to and transmigrations of the endothelium that lines the bone marrow sinuses by the monoclonal precursors, which contribute to the preferential trafficking of these cells in the bone marrow. The interaction between tumour cells and the bone marrow stromal cells promotes neoangiogenesis that is essential for myeloma growth and facilitate the lodging of new tumour cells in the bone marrow and their subsequent uncontrolled proliferation. This leads to the osteolytic activity responsible for the development of the bone lesions characteristic to myeloma [4,17].

This pathological process is orchestrated by cytokines, chemokines and growth factors. The neoangiogenesis evident in the bone marrow of subjects with myeloma is mediated by increased levels of basic fibroblast growth factor, vascular endothelial growth factor (VEGF), interleukin (IL)-lβ and tumour necrosis factor (TNF)-α. IL-6 is an essential growth factor for myeloma cells and promotes their survival. IL-1, VEGF, macrophage inhibitory factor (MIP) 1α, TNF-α, receptor activator of nuclear factor-κB (RANK) ligand and osteoprotegerin are agents mediating the osteoclastic activity that brings about the myeloma-associated osteolytic bony lesions [1,6,10,18].

Myeloma cells demonstrate cytogenetic abnormalities that may contribute to their proliferation and prolonged survival [1]. Myeloma cells do not have significant self-renewal potential and alone most probably cannot maintain the myeloma disease. Myeloma cells, like normal mature plasma cells express syndecan-1 (CD 138) cell surface antigen that is limited to terminally differentiated plasma cells originating of B lymphocyte lineage [19]. It is possible that the cells that maintain the oncogenic growth of myeloma are originally B lymphocytes which do not express CD 138. These cells are post-germinal center B cells, share monoclonal Ig gene sequences with myeloma cells, and subsequently differentiate into CD138+ myeloma cells. These particular B lymphocytes are probably already transformed and serve as neoplastic progenitor cells responsible for the perpetuation of myeloma [19]. This concept is supported by the evidence that plasmablastic myeloma with extramedullary involvement has an immunophenotypic profile and a morphologic pattern very similar to plasmablastic lymphoma, a post-germinal center B-cell/plasma cell neoplasm. At times, the differentiation between plasmablastic lymphoma and myeloma with extramedullary involvement must depend on such parameters as increased levels of monoclonal Ig molecules and osteolytic bone lesions that are diagnostic for myeloma [20,21].

This pathogenic background is significant for the treatment of myeloma. Current treatment targets myeloma cells, and assessment of response to therapy includes monitoring of the decline in bone marrow plasmacytosis and the decline in monoclonal Ig levels. Improvement in these parameters and in the clinical behavior of myeloma may be only temporary if the neoplastic progenitor B cells are not eradicated [19].

Myeloma or SP affecting the mouth and the jaws are uncommon. The mandible is more frequently involved than the maxilla and the bony lesions of both have a predilection for the posterior areas of the jaws [22,23]. It is estimated that in about 30% of subjects with either myeloma or with SP, the mouth and jaws may be involved [23], and oral lesions may be the primary manifestation [24,25]. The oral symptoms associated with myeloma or SP include osteolytic bone lesions, jaw pain, paraesthesia, burning mouth syndrome, amyloidosis of the oral soft tissues, haemorrhage, and an exophytic soft tissue growth [23-27].

Our patient was not aware of her HIV infection prior to our examination. Despite the fact that HIV infection is associated with increased frequencies of B cell lymphomas compared to the general population, there are no reports of a similar increase in the prevalence and incidence of myeloma tumors in relation to HIV infection [28,29]. However, the frequency of myeloma in HIV-seropositive subjects is increased compared to the general population [30]. Myeloma may be the first indicator leading to the diagnosis of HIV infection [31]. HIV-seropositive subjects are diagnosed with myeloma at a younger age, and have a more aggressive clinical course of their myeloma disease, compared to HIV-seronegative subjects [29,30,32,33].

The diagnosis of myeloma in HIV-seropositive subjects may not be straightforward because HIV infection and myeloma share some clinical and laboratory features including recurrent bacterial infections, anaemia, bone marrow plasmacytosis, polyclonal hypergamma-globulinemia and monoclonal gammopathy [28-30,34].

The pathogenic mechanisms that are associated with the increased frequency of myeloma in HIV-seropositive subjects compared to the general population are not well understood. However, the persistent polyclonal B cell proliferation related to HIV infection may eventually lead to clonal selection. The increased levels of interleukin 6 associated with HIV infection; and the clonal expansion of plasma cells caused by co-infection with other viruses (Epstein-Barr virus, human herpes virus-8) observed in HIV-seropositive subjects, are some possible mechanisms that are implicated in the evolution of B cell neoplasms and the development of myeloma in HIV-seropositive subjects [29,30,35].

## Conclusion

We presented a case of myeloma with an extensive destruction of the maxillofacial region and with intracranial involvement. The clinical picture was extreme and tragic.

This case report shows that myeloma should raise suspicion of HIV infection, and that myeloma in the setting of HIV infection can have an unusual aggressive clinical course.

## Consent

Written consent was obtained for the publication of this case report and any accompanying images. A copy of the written consent is available for review by the Editor-in-Chief of this journal.

## Competing interests

The authors declare that they have no competing interests.

## Authors' contributions

LF, JW, NHW, MB, JL, and EJR provided the study concept, and participated in its design and coordination. JW, NHW and MB performed the clinical work and case management. EJR performed histopathological studies. LF, JW, NHW and JL acquired data and performed the data analysis. LF, JL, MB and EJR were responsible for manuscript editing. LF, JW, NHW, MB, JL and EJR reviewed the manuscript. All authors read and approved the final manuscript.
